# Draft Genome Sequence of *Enterococcus faecium* PC4.1, a Clade B Strain Isolated from Human Feces

**DOI:** 10.1128/genomeA.00022-14

**Published:** 2014-02-06

**Authors:** Páraic Ó Cuív, Eline S. Klaassens, Wendy J. Smith, Stanislas Mondot, A. Scott Durkin, Derek M. Harkins, Les Foster, Jamison McCorrison, Manolito Torralba, Karen E. Nelson, Mark Morrison

**Affiliations:** a CSIRO Preventative Health Flagship Research Program, Queensland, Australia; b CSIRO Animal, Food and Health Sciences, St. Lucia, Queensland, Australia; c J. Craig Venter Institute, Rockville, Maryland, USA; d The Ohio State University, Columbus, Ohio, USA

## Abstract

*Enterococcus faecium* is commonly isolated from the human gastrointestinal tract; however, important intraspecies variations exist with relevance for host health and well-being. Here, we describe the draft genome sequence of *E. faecium* PC4.1, a clade B strain isolated from human feces.

## GENOME ANNOUNCEMENT

The facultative anaerobic bacterium *Enterococcus faecium* is among the early colonizers of the infant gut, where it has been shown to be transferred via the mother’s breast milk (1, 2). *E. faecium* is also one of the most abundant enterococcal species in the adult colon, where it likely plays an important role in maintaining host health and well-being, as has been suggested by human and animal studies (3–5). Significant intraspecies variations are known to exist; however, despite having an open pangenome (6), a recent comparative genomic analysis of *E. faecium* revealed the existence of only two distinct phylogenetic clades, termed clades A and B (7). Interestingly, most of the clinical and “healthy” isolates can be assigned to clade A and clade B, respectively, with clade B strains serving as important donors of DNA for clade A strains (8). Here, we describe the draft genome sequence of *E. faecium* PC4.1, isolated as part of the Australian Human Gut Microbiome Project, in order to provide new insights into the functional versatility of clade B strains.

*E. faecium* PC4.1 was isolated from a pooled fecal sample collected from healthy human subjects by plating aerobically on bile esculin azide agar. *E. faecium* isolates were distinguished from *Enterococcus faecalis* isolates by their β-galactosidase activity, as assessed on brain heart infusion agar supplemented with 5-bromo-4-chloro-3-indolyl-β-d-galactopyranoside. A 454 Life Sciences GS FLX system was used at the J. Craig Venter Institute (JCVI) to generate 2,811,160 bp of shotgun genomic DNA sequence at 20.7× coverage. Next, the Newbler Assembler version 1.1 was used to assemble the individual sequence reads, generating 78 contigs, with a contig N_50_ of 100.5 kb and with the largest contig assembled of approximately 272.2 kb. Finally, the JCVI prokaryotic annotation pipeline was used to annotate the DNA sequences.

The draft genome has a G+C content of 37.98% and contains 2,739 genes, including 2,695 protein-coding genes and 44 structural RNAs. As expected, several niche factors (9) were identified that likely contribute to its effective colonization and persistence in the human gut, including protein orthologs with predicted roles in binding host structural factors, including collagen, fibronectin, and nidogen. In addition, we also identified a putative lectin, which is consistent with the ability of enterococci to bind host sugar moieties (10). *E. faecium* PC4.1 encodes a candidate autolysin necessary for DNA release and biofilm formation (11); however, it does not encode the phosphotransferase system that is enriched in clinical isolates (7), nor does it encode the gelatinase and serine proteinase typically found in virulent enterococcal strains.

The draft *E. faecium* PC4.1 genome has provided an insight into the factors that support the ability of clade B isolates to colonize and persist in the human gut. Future studies should determine the genome phylogeny of putative pathogenic strains, including Crohn’s disease-associated strains (12, 13), and compare their functional potentials to those of healthy clade B isolates. The genome sequence of *E. faecium* PC4.1 and those of other healthy gut isolates will serve as valuable resources in this respect.

### Nucleotide sequence accession numbers.

This whole-genome shotgun project has been deposited at DDBJ/EMBL/GenBank under the accession no. ADMM00000000. The version described here is the first version, ADMM01000000.
